# Au, Pd and maghemite nanofunctionalized hydroxyapatite scaffolds for bone regeneration

**DOI:** 10.1093/rb/rbaa033

**Published:** 2020-08-27

**Authors:** Giovanna Calabrese, Salvatore Petralia, Claudia Fabbi, Stefano Forte, Domenico Franco, Salvatore Guglielmino, Emanuela Esposito, Salvatore Cuzzocrea, Francesco Traina, Sabrina Conoci

**Affiliations:** r1 Department of Chemistry Biology Pharmacy and Environmental Science, University of Messina, Piazza Pugliatti, 1, 98122 Messina, Sicilia, Italy; r2 Research and Development, Applied Chemical Works, Paternò, Catania, Italy; r3 Department of Chemical Science, Fin-Ceramica Faenza, Via Granarolo 177/3, Faenza 48018, Italy; r4 Istituto Oncologico del Mediterraneo Ricerca, Viagrande, Catania 95029, Italy; r5 Dipartimento Biomorf, Università degli Studi di Messina, Messina, Italy; r6 Rizzoli Orthopedic Institute, IRCSS, Bologna, Italy; r7 Distretto Tecnologico Micro e Nano Sistemi Sicilia, Catania, Italy

**Keywords:** bone regeneration, hydroxyapatite scaffold, tissue engineering, gold nanoroads, Pd nanoparticles, maghemite nanoparticles

## Abstract

Nanotechnology plays a key role in the development of innovative scaffolds for bone tissue engineering (BTE) allowing the incorporation of nanomaterials able to improve cell proliferation and differentiation. In this study, Mg-HA-Coll type I scaffolds (Mg-HA-based scaffolds) were nanofunctionalized with gold nanorods (Au NRs), palladium nanoparticles (Pd NPs) and maghemite nanoparticles (MAG NPs). Nanofunctionalized Mg-HA-based scaffolds (NF-HA-Ss) were tested for their ability to promote both the proliferation and the differentiation of adipose-derived mesenchymal stem cells (hADSCs). Results clearly highlight that MAG nanofunctionalization substantially improves cell proliferation up to 70% compared with the control (Mg-HA-based scaffold), whereas both Au NRs and Pd NPs nanofunctionalization induce a cell growth inhibition of 94% and 89%, respectively. Similar evidences were found for the osteoinductive properties showing relevant calcium deposits (25% higher than the control) for MAG nanofunctionalization, while a decreasing of cell differentiation (20% lower than the control) for both Au NRs and Pd NPs derivatization. These results are in agreement with previous studies that found cytotoxic effects for both Pd NPs and Au NRs. The excellent improvement of both osteoconductivity and osteoinductivity of the MAG NF-HA-S could be attributed to the high intrinsic magnetic field of superparamagnetic MAG NPs. These findings may pave the way for the development of innovative nanostructured scaffolds for BTE.

## Introduction

Tissue engineering (TE) and regenerative medicine (RM) are multidisciplinary research areas involving engineering, biology and material sciences to deliver innovative solutions to organ and tissue replacement. They aim to generate functional substitutes capable of repairing, sustaining or even improving damaged and diseased tissue functions [1]. TE conjugates three key components as follows: (i) scaffolds offering the support for cell growth and tissue formation; (ii) stem cells, typically seeded on the scaffolds, differentiating and creating the new tissue and (iii) growth factors regulating the differentiation and proliferation processes. This methodology can offer innovative solutions for bone repair, especially in the case of large bone defects, such as those originating from trauma, degenerative diseases, cancer resection and congenital deformities that are currently treated by invasive surgical procedures including autograft, allograft and xenograft [2]. Recent advances in the development of innovative biomaterials and local administration of bioactive molecules and growth factors have allowed overcoming the numerous limitations of grafting methods with a relatively high number of success rates and improved bone regeneration and tissue healing [3–5]. Bone tissue engineering (BTE), then, represents one of the most advanced methodologies for bone repairs. However, biomaterials for BTE require some crucial needs. In fact, they must be mechanically strong, chemically inert, biocompatible, biodegradable, osteoconductive, osteoinductive and capable of being osteointegrated at the implant site.

In this context, nanotechnologies can play a key role. They not only give the possibility to generate substrates similar to natural bone in terms of structure and size but also offer the opportunity for innovative nanofunctionalization to improve scaffold properties. In view of their nanoscale size, nanostructures exhibit unusual physical and chemical features that make them useful in a wide range of fields, from biomedical to electronics, optics and more [6–11]. Nanoparticles (NPs) of different materials such as ceramics, natural and synthetic polymers and metals have been widely studied as interesting potential candidates for BTE. More in details, ceramic nanomaterials such as hydroxyapatite (HA) are the most investigated NPs because their inorganic component is analogous to the natural bone matrix (essential to promote the integration of living bone tissue) and because they have an exceptional bioactivity, biocompatibility, osteoconductivity, osteoinductivity and biodegradability [12–15]. Recently, it has been demonstrated that the combination of HA NPs with electrospun chitosan matrix scaffolds promotes bone regeneration by supporting the adhesion and enhancing the proliferation of bone marrow mesenchymal stem cells both *in vitro* and *in vivo* [16]. Raucci *et al.* observed that the addition of HA NP fillers to matrix materials, such as gelatin, improves biological response of human mesenchymal stem cells (hMSCs) in terms of cell attachment, proliferation and osteoblastic differentiation [17]. In addition, many studies have been addressed to the combination of HA NPs with other polymers, such as polycaprolactone [18], poly(lactic-co-glycolic acid) and poly(ethylene glycol) [19] for the production of composite scaffolds aiming at improving osteogenesis.

The integration of NPs in BTE biomaterials has been widely explored attracting great attention due to their significant potential to enhance both mechanical and biological properties, such as osteointegration, osteoconduction and osteoinduction [20]. In this context, metallic NPs have been exploited combined with scaffolds due to their several innate properties, such as antimicrobial activity, mechanical strength and their capability to stimulate osteogenic and, in some cases, angiogenic activities [21, 22]. Moreover, metal NPs have also the advantage of being safer, stable, cheap and less probable to stimulate a strong immune response than other materials [23]. More in details, gold NPs (Au NPs) have been extensively investigated for TE and RM applications, due to their advantages of optical properties, superior biocompatibility, easy synthesis in different shapes and sizes and surface functionalization [24–28]. Several studies reported that Au NPs are beneficial for BTE, demonstrating osteogenic differentiation of stem cells and increased mineralization [29]. Moreover, further literature evidences have suggested that Au NPs could induce stem cell differentiation into osteoblasts in a size and shape dependent manner. Li *et al.*, in their study, demonstrated that Au nanostructures with diameters between 40 and 70 nm greatly improve the osteogenic differentiation of hMSC [30]. In addition, other researchers showed that Au NPs cytotoxicity is both associated to the shape, size and concentration and to the cell line utilized [31–33]. Au nanoflowers, nanoprisms and nanostars induce toxic effects on the cells only at high concentrations, whereas Au nanorods and nanospheres, with smaller sizes (from 1 to 45 nm), are cytotoxic already at low concentrations probably due to their affinity to aggregate inside the cell [34].

Among metal NPs, palladium NPs (Pd NPs) have been lately investigated because they display remarkable antimicrobial, electrical, catalytic and photosensitive properties due to the particle size and shape. Balaji *et al.* evaluated the effects of Pd NPs on nanocomposite scaffolds, consisting of reduced graphene oxide (rGO) functionalized with polypyrrole (PPy) (Pd/PPy/rGO), and demonstrated that Pd NPs ensure prevention from colonizing, adhering and forming microbial biofilms on the substrates of these biomaterials [35].

Superparamagnetic iron oxide nanoparticles (SPIONs), such as magnetite (Fe_3_O_4_) or its oxidized form maghemite (MAG) (γ-Fe_2_O_3_), have also stimulated a relevant interest, especially in the BTE field, due to their significant magnetic, chemical, thermal and mechanical properties [36]. At this regard, several studies examined the effects of SPIONs on bone repair. Meng *et al.* suggested that electrospun combined scaffold, composed of superparamagnetic γ-Fe_2_O_3_ NPs, HA NPs and polylactic acid, under an applied external magnetic field, accelerates the proliferation and differentiation of osteoblast cells *in vitro* and the formation and remodeling of new bone tissue *in vivo* [37, 38]. Further, it has been demonstrated that SPION aggregations induced by magnetic fields are able to promote the osteoblast differentiation from primary mouse bone marrow cells and to improve bone repair [39, 40].

Based on the above considerations, in this study, the integration of gold nanorods (Au NRs), Pd NPs and MAG NPs into Mg-HA-Coll type I scaffold (Mg-HA-based scaffold) was carried out. The prepared nanofunctionalized Mg-HA-based scaffolds (NF-HA-Ss) were characterized and tested in their osteoconductive and osteoinductive properties for both proliferation and differentiation toward hADSCs. The results are presented and discussed.

## Materials and methods

### Chemicals

All chemicals were obtained from commercial sources at the highest possible purity and were used as received. All solvents used were spectrophotometric grade. Milli-Q-grade water was used in all preparations.

### Mg-HA-based scaffold structure

**Figure 1:**
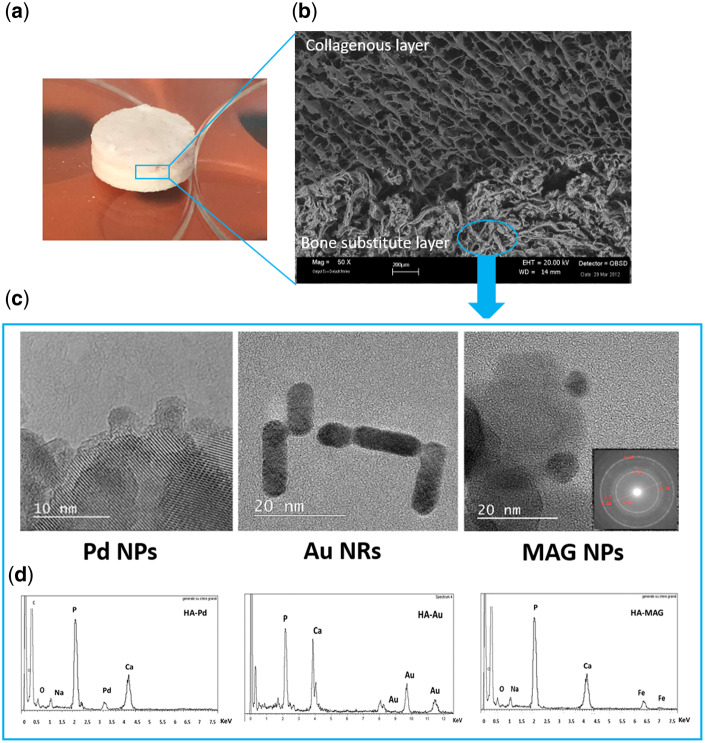
(**A**) Mg-HA-based scaffold (Mg-HA-Coll type I), (**B**) representative SEM image of Mg-HA-based scaffold section, (**C**) representative TEM images of Pd NPs, Au NRs and MAG NPs (inset shows the SAED pattern) and (**D**) EDX analysis of NF-HA-Ss.

Mg-HA-based scaffolds were provided by Fin-Ceramica Faenza SpA (Faenza, Italy) and they were developed according to the fabrication process previously reported [41, 42]. In briefly, these scaffolds have a cylindrical shape (8 mm in diameter and 5 mm high) and are composed of a bilayer structure (Fig. 1A), in which the top layer (3 mm in depth) consists of equine type I collagen (Coll) deposed on a second layer (2 mm depth) made of a mineralized blend of type I Coll (30%) and Mg-HA (70%).

The morphological and microstructural analysis of the Mg-HA-based scaffold was executed by Scanning Electron Microscopy (SEM) performed on an SEM-LEO 438 VP (Carl Zeiss AG, Oberkochen, Germany). The samples were sputter coated with gold prior to examination.

### Nanofunctionalization of Mg-HA-based scaffold

The Mg-HA-based scaffolds were nanofunctionalized with nanostructures using the following procedure as reported.

#### Au-NF-HA-Ss (HA_Au)

The Au NRs were formed by standard synthesis based on reduction of gold (III) chloride trihydrate by sodium borohydride usingCTAB (hexadecyltrimethylammonium bromide) as a surfactant. In detail, 15 ml aliquot of HAuCl_4_ 5 × 10^−4^ M was heated, using a temperature-controlled oil bath, in a flask at 75°C and, under stirring, the same volume of a CTAB solution of 5 × 10^−1^ M, under the previous conditions, was added. After 10 min, 1 ml of a silver nitrate solution (1 × 10^−1^ M) was added, later a volume of 1 ml of an l-ascorbic acid solution (1.0 M) and a volume of 10 µl of freshly prepared aqueous solution of sodium borohydride solution (1 × 10^−2^ M) were added, the mixture appears instantly dark blue and was stirred at room temperature for 30 min in darkness condition. After that the Au NPs were separated by centrifugation for 3 min at 12 000 rpm, then the blue solid Au NRs washed with deionized water. The gold NA-HA-Sss (HA_Au) were prepared by dipping in 10 ml of Au NPs aqueous solution overnight at room temperature. After that, the HA_Au derivatized scaffolds were washed by deionized water and dried by nitrogen flow.

#### Pd-NF-HA-Ss (HA_Pd)

Mg-HA-based scaffolds were dipped in 10 ml of Palladium acetylacetonate (Pd(acac)_2_) de-aerated ethanolic solution (concentration 20 mg/100 ml) in a quartz tools. An aliquot (100 μl) of acetone was added to the reaction as a photosensitizer. After degassing with Ar for 15 min, the reaction was irradiated with 4 UV lamps (254 nm, 16 W) for 5 min. The brown HA_Pd scaffolds were rinsed with deionized water and dried with nitrogen flow.

##### MAG-NF-HA-Ss (HA_MAG)

γ-Fe_2_O_3_ NPs were synthetized using the following procedure. FeCl_3_ and FeCl_2_ powder were dissolved in HCl of 1 M to achieve a final concentration of 1 M FeCl_3_ and 2 M FeCl_2_. Then, ammonia solution of concentration 2 M was dropped into this solution with vigorous stirring at room temperature for 3 h. The darkness precipitate was then collected by filtration and rinsed three times with deionized water. Mg-HA-based scaffolds were then dipped in aqueous solution (10 ml) of MAG (γ-Fe2O3) NPs, overnight at room temperature. Finally, the red-brown HA_MAG scaffolds were rinsed for three times with deionized water and dried with nitrogen flow.

The NF-HA-Ss were sterilized with UV light overnight before the *in vitro* tests.

### TEM, EDX and UV-Vis analyses

Transmission electron microscopy (TEM) analysis was performed using the bright field in conventional TEM parallel beam mode. An ATEMJEOL JEM-2010 equipped with a 30-mm^2^ window energy-dispersive X-rays (EDX) spectrometer was used. UV-Vis spectroscopy and absorption spectra were recorded with a Jasco V-560 homemade photoreactor equipped with UV light (four lamps, 16 W at 254 nm).

### hADSCs culture

hADSCs line used in this study was purchased from Thermo Fisher Scientific (NYSE:TMO; Waltham, MA) and grown in MesenPRO RS™ medium (Thermo Fisher Scientific, NYSE:TMO) up to 70–80% confluence.

### hADSCs osteogenic differentiation on nanofunctionalized scaffolds

For osteogenic differentiation, 1 × 10^6^ hADSCs suspended in 50 µl of MesenPRO RS™ medium were gradually drop seeded onto the bone layer of bilayer scaffold and incubated for 4 h at 37°C. Successively, MesenPRO RS™ medium was added and (day 0) replaced with osteogenic differentiation medium (StemPRO™ osteogenesis differentiation kit, Thermo Fisher Scientific, NYSE:TMO), after 24 h. The osteogenic differentiation medium was wholly substituted twice a week. Each scaffold has been analyzed after 24 days of osteogenic induction.

### Histological analysis on hADSCs-nanofunctionalized scaffolds

NF-HA-Ss with hADSCs were fixed in 4% PFA (Paraformaldeide), dehydrated, embedded in paraffin and cut into 5-µm-thick sections. Sections were mounted on slides and treated for histological staining. After deparaffinization and rehydration, alternate sections were stained with either hematoxylin and eosin (H&E) or Alizarin Red S (Panreac, Castellar del Valles, Barcellona, Spain) as described previously [43]. For Alizarin Red S staining, the solution was prepared according to the manufacturer protocol, added to the sections and incubated for 5 min. After, the sections were washed several times to get rid of the excess of the staining solution and finally, mounted. The stained sections were analyzed by using a Leica DMI 4000B microscope (Leica Microsystems Srl, Milano, Italy). At least three sections/scaffold were analyzed.

### Cell proliferation analysis through DAPI staining on hADSCs-nanofunctionalized scaffolds

For 4',6-diamidino-2-phenylindole (DAPI) staining, ‘hADSCs-nanofunctionalized scaffolds’ were fixed in 4% PFA, dehydrated, embedded in paraffin and cut into 5-µm-thick sections. Sections were permeabilized in 0.3% Triton X-100 for 10 min, washed in phosphate-buffered saline (PBS) and the nuclei stained with DAPI (1:5000) in PBS for 5 min. Slides were mounted in fluorescent mounting medium (PermaFluor, Thermo Scientific), and digital images were acquired using a Leica DMI4000B fluorescence microscope. Three images from each scaffold were analyzed. Cell count analysis has been performed using Fiji image J recognition software.

### Immunohistochemical analysis on hADSCs-nanofunctionalized scaffolds

hADSCs-nanofunctionalized scaffolds were fixed in 4% PFA, embedded in paraffin cut into 5-µm-thick sections, mounted on slides and processed for immunohistochemical analysis. After deparaffinization and rehydration, the slides were permeabilized with 0.4% Triton-X100, blocked with 4% bovine serum albumin and incubated with the rabbit polyclonal primary antibodies: antiosteocalcin (BGLAP; 1:50, LSBio, Seattle, WA) and antiosteonectin (SPARC; 1:50, LSBio) overnight at 4°C. The next day, slides were incubated for 2 h at room temperature with Alexa Fluor antirabbit 568 secondary antibody (1:2000, Life Technologies Italia, Monza, Italy), washed, counterstained with DAPI (1:5000) and mounted with PermaFluor aqueous mounting medium (Thermo Scientific). Control of immunostaining specificity was performed omitting the primary antibody.

### Statistical analysis

Data were analyzed either as raw data or as mean ± standard error (SE), as appropriate. Differences between nanofunctionalized scaffolds were evaluated by using one-way analysis of variance (ANOVA) with post-hoc Bonferroni test, where appropriate. *P* < 0.05 was considered to be significant.

## Results and discussion

Figure 1 reports the details of the Mg-HA-based scaffold investigated in the present study. It consists of bioactive Mg-doped hydroxyapatite (Mg-HA) nanocrystals integrating type I Coll organic compound (Fig. 1A). The morphological and microstructural analysis of the scaffold was performed by SEM. Representative image of the scaffold section is shown in Fig. 1B. Two main strata are present: a top layer (3 mm in depth) composed by 100% type I Coll (collagenous layer) and bottom layer (2 mm in depth) made of 70% of Mg-HA and 30% of type I Coll (bone substitute layer) mimicking a bone-like tissue [42]. The collagenous layer shows well-interconnected pores with diameters up to 300 µ and evident large channel around 600 µm. The porosity distribution goes from 16 to 180 µm (with large pores excluded from the analysis). The biomineralized collagen fibers create a highly fibrous structure and present a homogeneous distribution of pores without any preferential alignment of the mineralized collagen fibers. The bone substitute layer exhibits well-interconnected pores (diameters from 20 to 175 µm) with 50% of pores have diameters ranging from 50 to 80 µm. The porosity of the bone substitute layer resulted 89 ± 7%.

The above described Mg-HA-based scaffolds were nanofunctionalized with nanostructures of Au NRs, MAG NPs and Pd NPs, respectively. The structural features of these nanostructures were examined by TEM analysis (Fig. 1C). A diameter mean value of 4 ± 2 nm and length of 15 ± 5 nm was found for the Au NRs with a UV-Vis optical absorption spectrum showing the typical plasmon absorption band centered at 530 nm (data not showed). Pd NPs show a diameter mean value of 3.4 ± 1 nm and MAG NPs exhibit nanosized iron oxide species with a diameter mean value of about 10 nm. The diffraction analysis of MAG NPs (data not shown) indicates the diagnostic d-spacing values for the MAG. Following the d-spacing values, percentage intensities have been found as follows: 2.95 Å (28%), 2.54 Å (100%), 2.09 Å (20%), 1.73 Å (10%), 1.64 Å (25%) and 1.47 Å (42%). According to the literature data [44], these values confirm the formation of the MAG structure.

Morphological and microstructural analyses of the NF-HA-Ss were assessed by both SEM and EDX analyses. The SEM inspection does not show any modifications of the structural features upon the nanofunctionalization (see Supplementary Fig. SI-1). The EDX analysis confirms the presence of nanomaterials, with diagnostic peaks recorded in the range 3.0–3.2 keV for Pd NPs, 8.0 and 11.5 keV for the Au NRs and, finally, 6.4 and 7.0 keV for MAG NPs nanofunctionalization. The EDX investigation indicates that the amount of chemical species (Au, Pd and Fe) ranging from 1 to 5% w/w and they are spread out on surface without presence of localized higher concentration zone.

**Figure 2:**
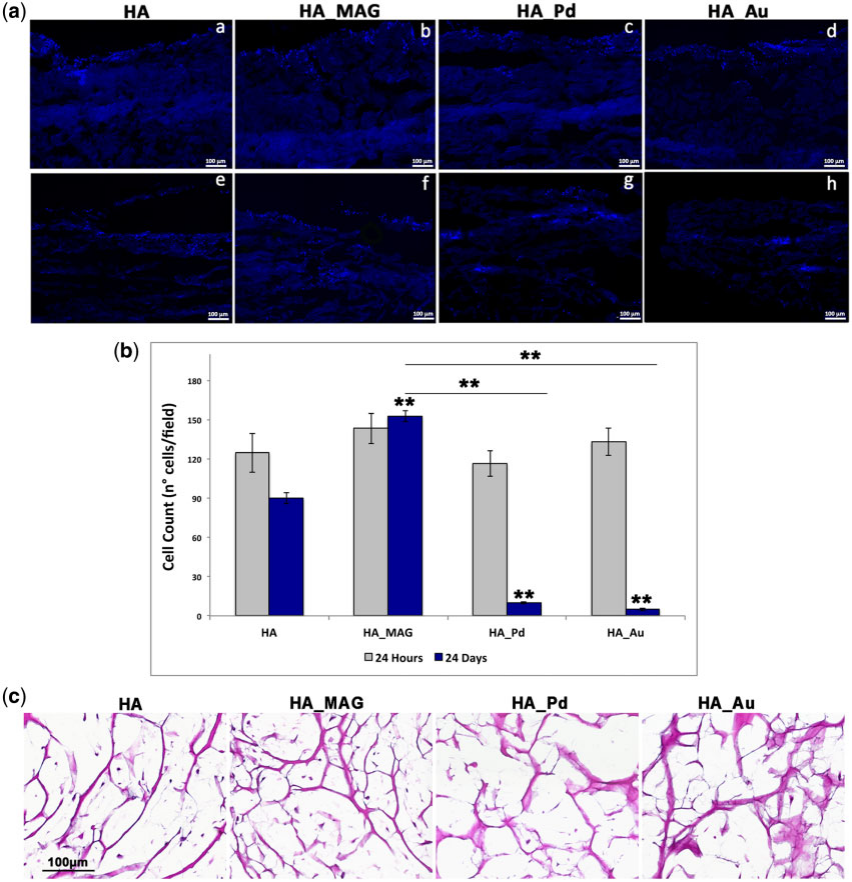
(**A**) Representative DAPI images after 24 h and 24 days of osteogenic induction on scaffolds: HA (control—Mg-HA-based scaffold), HA_MAG, HA_Pd and HA_Au. (**B**) Cell count from DAPI staining after 24 hours and 24 days of osteogenic differentiation. ANOVA test *P* values is reported (*P* < 0.0001) and (**P < 0.01) indicates significant differences between scaffolds as reported by the Bonferroni post-hoc test; (**C**) representative images of H&E staining on NF-HA-Ss maintained for 24 days in osteogenic medium: HA (control), HA_MAG, HA_Pd and HA_Au scaffolds. Scale bars: 100 μm.

To evaluate the capability of seeded cells to adhere, penetrate and propagate into the NF-HA-Ss (‘Osteoconductive ability’), hADSCs were cultured in presence of osteogenic differentiation medium for 24 days. DAPI staining on hADSCs-nanofunctionalized scaffold sections at two specific time points, 24 h and 24 days, were performed and cells number counting evaluated compared with the control. Results are reported in Fig. 2A and B. It can be noticed that data collected at 24 h after cells seeding did not display any significant statistical difference in the number of cells present on the NF-HA-Ss (Fig. 2A, a–d) corresponding to about 125 cells/field. On the contrary, after 24 days of osteogenic differentiation, a different behavior of the NF-HA-Ss was observed (Fig. 2A, e–h). In particular, the control (Mg-HA-based scaffold) shows an adherent cell number of 90 ± 4 cells/field, whereas HA_MAG, HA_Pd and HA_Au nanofunctionalized scaffolds exhibit a cell number of 152 ± 4, 10 ± 1 and 5 ± 1 cells/field, respectively. These data indicate that in case of Pd and Au nanofunctionalization, the cell numbers decrease of 89% and 94% compared with the control, indicating an inhibition of cell proliferation. On the contrary, HA_MAG scaffold exhibits a relevant increasing of cell number of 70% compared with the control, clearly highlighting that the MAG nanofunctionalization substantially improves the cell proliferation on the scaffold. These findings were confirmed with H&E staining as reported in Fig. 2C. It can be noticed a very low presence of cells in the whole HA_Pd and HA_Au scaffolds compared with the control, whereas HA_MAG scaffolds exhibit very high ability of the hADSCs to penetrate and invade the whole scaffold and proliferate. Results collected by both DAPI and H&E analyses undoubtedly indicate that the MAG nanofunctionalization considerably increases the osteoconductive ability of Mg-HA-based scaffolds compared with the control, Pd and Au nanofunctionalization, in terms of cell adhesion, penetration and proliferation.

**Figure 3:**
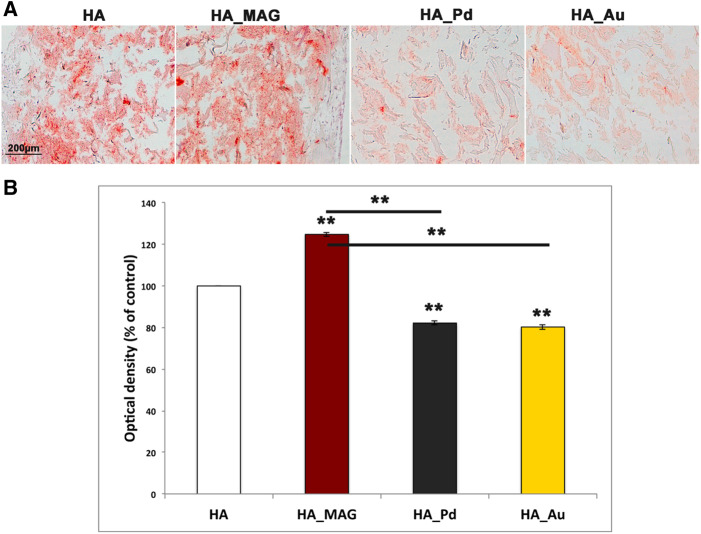
(**A**) Representative images of Alizarin Red S staining collected after 24 days of osteogenic differentiation: HA (control—Mg-HA-based scaffold), HA_MAG, HA_P and HA_Au scaffolds. Scale bars: 200 μm. (**B**) Optical densities quantification of Alizarin Red S staining images (mean and SD obtained on three images/scaffold).

The second part of our study was devoted to the assessment of the osteoinductive properties of the NF-HA-Ss. This was achieved by analyzing both the matrix mineralization density and the expression of specific bone immunomarkers upon hADSCs osteogenic differentiation after 24 days. More in details, the matrix mineralization was evaluated by Alizarin Red S staining. Data are reported in Fig. 3. It can be noticed the presence of relevant calcium deposits in case of HA_MAG scaffold (125 ± 1% respect to the control, the naked Mg-HA-based scaffold), whereas both HA_Pd and HA_Au show very low presence of calcium deposits (82 ± 1% for HA_Pd and 80 ± 1% for HA_Au respect to the control). These data suggest that the MAG nanofunctionalization leads to a relevant improvement of bone augmentation, whereas the treatments with both Au and Pd NPs induce a reduction of calcium with consequent lower bone matrix.

**Figure 4:**
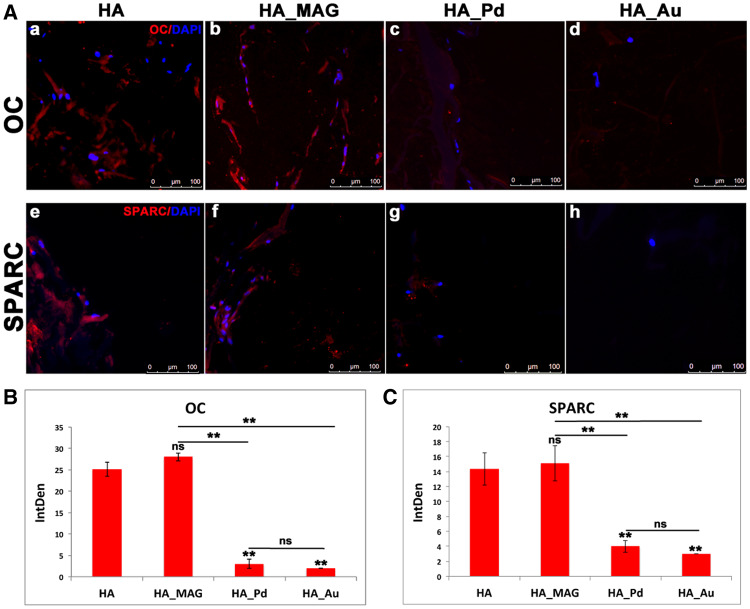
(**A**) Immunohistochemical analyses of OC marker: (a) HA (control—Mg-HA-based scaffold), (b) HA_MAG scaffold, (c) HA_Pd scaffold and (d) HA_Au scaffold and SPARC marker: (e) HA (control—Mg-HA-based scaffold), (f) HA_MAG scaffold, (g) HA_Pd scaffold and (h) HA_Au scaffold. Scale bars: 100 μm. (**B**, **C**) Integrated density (IntDen) of osteogenic markers, OC and SPARC. One-way ANOVA *P* values are reported. Symbols above bars indicate statistically significant differences (*P < 0.05, **P < 0.01, ns, not significant) in the Bonferroni post-hoc tests. Three images for scaffolds have been used.

The immunohistochemical analyses of specific osteogenic markers consisting in osteonectin (SPARC) for bone mineralization and osteocalcin (OC) for mature osteoblasts is reported in Fig. 4. The data highlight positive signals for HA_MAG scaffold (Fig. 4A, b and f) and the control (Fig. 4A, a and e), whereas negligible signals were detected in HA_Pd and HA_Au scaffolds (Fig. 4A, c, g and d, h).

According to the immunofluorescence images (Fig. 4A), the semi-quantitative analysis of the OC and SPARC markers showed a significant and comparable integrated densities only for HA and HA_MAG, whereas negligible fluorescence intensities were detected for both HA_Pd and HA_Au.

These findings prove that whereas both Pd and Au nanofunctionalizations inhibit the osteoinductive properties of the Mg-HA-based scaffold, the HA_MAG nanofunctionalization substantially improve the hADSCs osteogenic differentiation.

These results clearly suggest that nanofunctionalization with Pd and Au metal nanostructures induce a reduction of both osteoconductive and osteoinductive properties of the Mg-HA-Coll type I scaffold. The hypothesis of cytotoxic effects of both nanomaterials against hADSCs can be proposed. In fact, it has been already reported that Pd nanomaterials show anticancer activity due to cellular toxicity through reactive oxygen species formation inducing autophagy and cell apoptosis [29, 45]. They were also relevant for antimicrobial activity [46], so that they have been studied as materials for biofilm prevention and destruction [35]. These data well support our results of cytotoxicity found against hADCSs. Concerning Au NPs, several studies report different properties in TE and RM mainly depending from size and shape of the gold nanostructures [47]. In particular, NPs with smaller sizes, such as nanorods and nanospheres ranging from 1 to 45 nm were found to be more toxic than larger conformations, such as Au nanoflowers, nanoprisms and nanostars attributing this finding to the tendency of smaller NPs to aggregate inside the cell [34]. Based on literature data, as the NRs here investigated are dimensionally in the range of 1–45 nm, we can propose that the observed reduction of osteoconductivity and osteoinductivity versus hADSCs can be attributed to some toxic effect due to the specific size and shape of our Au nanostructures. These findings certainly stimulate further investigations exploring the effects of several Au nanostructures of different size/shape and concentration.

In contrast to the results above discussed on both Au and Pd NPs, the data collected on the Mg-HA-Coll type I scaffold nanofunctionalized with MAG NPs show an improvement of both proliferation and differentiation of hADCs. This can be likely attributed to the magnetic properties of MAG (γ-Fe_2_O_3_). Although there are several types of iron oxides in nature, only magnetite (Fe_3_O_4_) and MAG (γ-Fe_2_O_3_) are appealing for biomedical applications due to their high magnetic moments, low toxicity and chemical stability in physiological environments [48]. Some studies well demonstrate that the iron oxide combined with HA crystals enhanced proliferation activity of osteoblasts [49], being the magnetic material a promoting factor for bone regeneration especially when the therapy is combined with magnetic stimulation [50]. However, γ-Fe_2_O_3_ NPs are reported to be better material for bone regeneration compared with Fe_3_O_4_ due its superparamagnetic properties that enhance the cell growth rate [51]. Considering the above literature reports, we can reasonably attribute the excellent improvement of osteoconductivity and osteoinductivity of the Mg-HA-based scaffold nanofunctionalized with MAG NPs to the intrinsic magnetic field of these superparamagnetic NPs. Further investigation will be performed to gain more insight on this aspect by correlating size/shape and concentration of MAG NPs with their intrinsic magnetic field and possibly the assessment on the bone regeneration performance also with the application of external magnetic fields.

## Conclusions

In this study, Mg-HA-Coll type I scaffolds were functionalized with Au NRs, Pd NPs and MAG NPs obtaining new NF-HA-S scaffolds. The structural features of these nanosystems show mean diameter of 4 ± 2 nm and length of 15 ± 5 nm for Au NRs, a diameter mean value of 3.4 ± 1 nm for Pd NPs and 10 nm for MAG NPs, respectively. NF-HA-S scaffolds were investigated in their ability to promote both proliferation and differentiation of hADSCs. Results for the osteoconductive properties clearly highlight that the functionalization with MAG NPs substantially improve the cell proliferation achieving + 70% compared with the HA naked scaffold (the control). On the contrary, the HA scaffold functionalized with both HA NPs and Pd NPs show cell growing inhibition with a decreasing of 94% and 89% of cell proliferation compared with the control. Similar behavior was observed also for the osteoinductive properties assessed by analyzing both the matrix mineralization density (Alizarin Red staining) and the expression of SPARC (specific for bone mineralization) and OC (specific for mature osteoblasts) markers. The findings highlight an improvement of differentiation for MAG nanofunctionalization proved by the increasing of calcium deposits of 25% more respect to the control and the presence of both SPARC and OC markers. On the contrary, both the HA_Au and HA_Pd scaffolds show a decreasing of calcium deposits of about 20% of calcium deposits and negligible presence of the osteogenic markers. These findings can be attributed to some cytotoxic activity from both Pd NPs and Au NRs. Concerning the relevant improvement of both osteoconductivity and osteoinductivity observed for the Mg-HA-based scaffold nanofunctionalized with MAG NPs, this can be likely attribute to the superparamagnetic properties of MAG (γ-Fe_2_O_3_) that has been already reported to enhance and promote cell growth rate. However, also in this case, the result obtained stimulates further investigations to gain more insight on the correlation between the physical properties of MAG NPs (size/shape, concentration, intrinsic magnetic field value) and the bone regeneration.

The results presented in this study pave the way for the development of innovative nanostructured scaffolds for the design of effective biomaterials in bone regeneration.

## Funding

This work was supported by PON—BONE++, Sviluppo di Micro e Nanotecnolgie per la Predittività, la Diagnosi, la Terapia e i Trattamenti Rigenerativi delle Alterazioni Patologiche dell’Osso e Osteo-Articolari (No. ARS01_00693).

*Conflict of interest statement*. None declared.

## Supplementary Material

rbaa033_Supplementary_Data
