# Supplementary material for: Au, Pd and maghemite nanofunctionalized hydroxyapatite scaffolds for bone regeneration
Source: Regen Biomater. 2020 Aug 27;7(5):461–9. doi: 10.1093/rb/rbaa033 (PMC7597806; doi:10.1093/rb/rbaa033)
Supplement: rbaa033_Supplementary_Data [file rbaa033_supplementary_data.docx]

**SUPPORTING INFORMATION**

**Au, Pd and Maghemite Nanofunctionalized Hydroxyapatite Scaffolds for Bone Regeneration**

Giovanna Calabrese^1Φ^, Salvatore Petralia^2Φ^, Claudia Fabbi^3^, Stefano Forte^4^, Domenico Franco^1^, Salvatore Guglielmino^1^, Emanuela Esposito^1^, Salvatore Cuzzocrea^1^, Francesco Traina^5,6*^ and Sabrina Conoci^1,7*^.


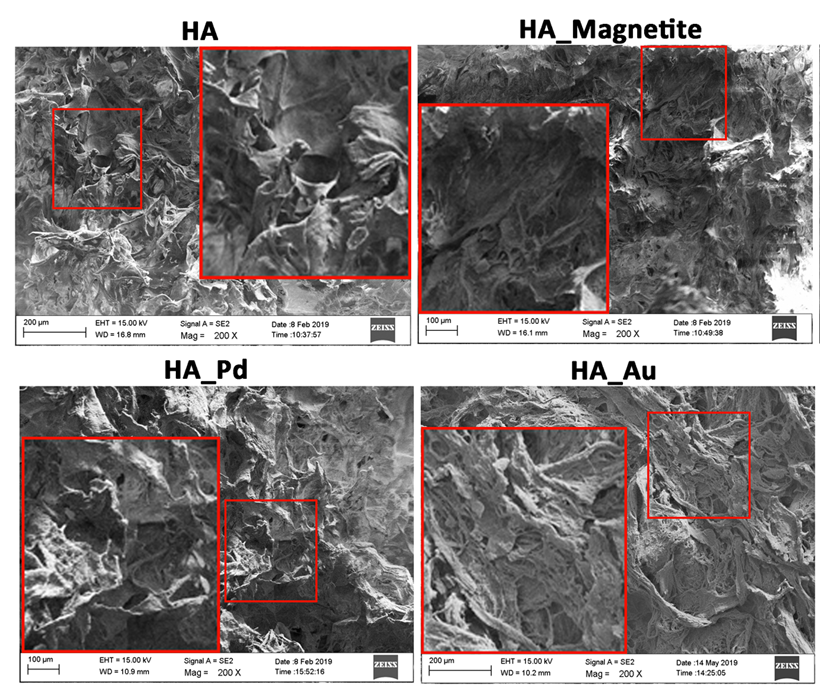


**Figure SI-1**. Representative SEM images of Pd NPs, Au NRs, and MAG NPs
